# Involvement of a BH3-only apoptosis sensitizer gene *Blm-s* in hippocampus-mediated mood control

**DOI:** 10.1038/s41398-022-02184-6

**Published:** 2022-09-26

**Authors:** Pei-Hsin Huang, Tsung-Ying Yang, Chia-Wei Yeh, Sheng-Min Huang, Ho-Ching Chang, Yun-Fen Hung, Wen-Chia Chu, Kuan-Hung Cho, Tzu-Pin Lu, Po-Hsiu Kuo, Li-Jen Lee, Li-Wei Kuo, Cheng-Chang Lien, Hwai-Jong Cheng

**Affiliations:** 1 Graduate Institute of Pathology, College of Medicine, National Taiwan University, 100 Taipei, Taiwan; 2 Department of Pathology, National Taiwan University Hospital, 100 Taipei, Taiwan; 3 Institute of Neuroscience, College of Life Sciences, National Yang Ming Chiao Tung University, 112 Taipei, Taiwan; 4 Institute of Biomedical Engineering and Nanomedicine, National Health Research Institutes, 350 Miaoli, Taiwan; 5 Institute of Molecular Biology, Academia Sinica, 115 Taipei, Taiwan; 6 Department of Public Health & Institute of Epidemiology and Preventive Medicine, College of Public Health, National Taiwan University, 100 Taipei, Taiwan; 7 Department of Psychiatry, National Taiwan University Hospital, 100 Taipei, Taiwan; 8 Graduate Institute of Anatomy and Cell Biology, College of Medicine, National Taiwan University, 100 Taipei, Taiwan; 9 Institute of Brain and Mind Sciences, College of Medicine, National Taiwan University, 100 Taipei, Taiwan; 10 Institute of Medical Device and Imaging, College of Medicine, National Taiwan University, 100 Taipei, Taiwan; 11 Brain Research Center, National Yang Ming Chiao Tung University, 112 Taipei, Taiwan

**Keywords:** Molecular neuroscience, Depression

## Abstract

Mood disorders are an important public health issue and recent advances in genomic studies have indicated that molecules involved in neurodevelopment are causally related to mood disorders. BLM-s (BCL-2-like molecule, small transcript isoform), a BH3-only proapoptotic BCL-2 family member, mediates apoptosis of postmitotic immature neurons during embryonic cortical development, but its role in the adult brain is unknown. To better understand the physiological role of *Blm-s* gene in vivo, we generated a *Blm-s*-knockout (*Blm-s*^*−/−*^) mouse. The *Blm-s*^*−/−*^ mice breed normally and exhibit grossly normal development. However, global depletion of *Blm-s* is highly associated with depression- and anxiety-related behaviors in adult mutant mice with intact learning and memory capacity. Functional magnetic resonance imaging of adult *Blm-s*^*−/−*^ mice reveals reduced connectivity mainly in the ventral dentate gyrus (vDG) of the hippocampus with no alteration in the dorsal DG connectivity and in total hippocampal volume. At the cellular level, BLM-s is expressed in DG granule cells (GCs), and *Blm-s*^*−/−*^ mice show reduced dendritic complexity and decreased spine density in mature GCs. Electrophysiology study uncovers that mature vGCs in adult *Blm-s*^*−/−*^ DG are intrinsically more excitable. Interestingly, certain genetic variants of the human *Blm* homologue gene (*VPS50*) are significantly associated with depression traits from publicly resourced UK Biobank data. Taken together, BLM-s is required for the hippocampal mood control function. Loss of BLM-s causes abnormality in the electrophysiology and morphology of GCs and a disrupted vDG neural network, which could underlie *Blm-s*-null-associated anxiety and depression.

## Introduction

Mood disorders such as depression and anxiety are common public health problems with a lifetime prevalence of 4–29%, and both rank among the top ten causes of disability [1, 2]. Mood disorders are heterogeneous diseases, involving multiple factors, including neural circuits, neuromodulators, immune function, and genetic factors [3–7]. The mood is governed by distributed brain circuitry, which includes the hippocampus, amygdala, hypothalamus-pituitary axis, ventral tegmental area, nucleus accumbens, habenula, and medial prefrontal cortex [8]. Dysfunction in any component of this basic emotion-control circuitry results in a pathological state of mood [9]. Evidence from neuroimaging of human patients and animal models has identified activities of some brain regions as the signature of depression or anxious mood. Particularly, the hippocampus, as a key limbic structure, is functionally important in mood control. A reduction in hippocampal volume is frequently observed in humans with major depression, and the magnitude of decreased hippocampal size positively correlates with the frequency of depression episodes [10, 11]. The hippocampus also constitutes an important node connected to the default mode network (DMN), a type of resting-state network (RSN) that includes the precuneus/posterior cingulate cortex (PCC), medial prefrontal cortex (mPFC), inferior parietal and medial temporal cortex [12, 13]. RSN shows synchronous low-frequency oscillation in the resting brain but deactivates when attention-demanding task performance is initiated. It is thought to function in the organization of preplanned reflective behaviors that are necessary for our existence in a complex world. Altered RSN patterns are frequently associated with neuropsychological disorders, including schizophrenia, autism spectrum disorder, post-traumatic stress disorder, attention deficit hyperactive disorder, temporal lobe epilepsy, depression, Parkinson's disease, and Alzheimer’s disease [14]. Changes in the hippocampal resting-state connectivity have been related to declined neurocognitive functions [15] and major depressive disorder [16, 17] in humans. Interestingly, several RSNs have also been detected in anesthetized rodent brains, indicating resting-state activity as an evolutionarily preserved feature [18–20]. Studies of resting-state connectivity in animal models such as rodents can therefore provide clues about the dysregulated circuitry underlying human mood disorders.

Depression and anxiety might share the same etiology since both are marked by impaired cognition and respond to the same treatment. Although multiple neural, cellular, and molecular mechanisms have been proposed to explain mood control, no unified theory that integrates all information from studies of micro-, meso-, and macro-scales could be proposed to explain the pathophysiology of mood disorders [7]. The lack of thorough understanding of mood disorders is also reflected in the fact that more than one-third of depression patients are resistant to current antidepressant treatment [21–23]. Despite all these facts, genetic studies clearly indicate that mutations in certain ‘risk genes’ enhance the probability to develop mood disorders, pointing to a strong genetic component in the etiology of mental illnesses [24, 25]. The heritability of major depressive disorder and anxiety is estimated to be ~7–12% by SNP-based (h^2^) studies [26, 27] and ~30–43% via twin and family studies [28, 29]. In addition, accumulative genome-wide association studies have led to the identification of 102 independent genetic variants and 269 genes associated with depression [26]. Here, in line with the view that genetic factors are critical determinants of mood control, we present our findings to show that deficit of a BCL-2 (B-Cell Lymphoma 2) family member BLM-s (BCL-2 like molecule, short transcript isoform) causes anxiety- and depression-like behavior.

BLM-s is a BH3-only apoptosis sensitizer/derepressor of the BCL-2 family, which consists of >40 members harboring the evolutionarily highly conserved BH domain and plays important roles in the regulation of cell death under various physiological or pathological conditions [30, 31]. BLM-s is an isoform encoded by the *Blm-l* gene (also known as *mVps50* [32], *Syndetin* [33], *mVps54L* [34], and *CCDC132* [35]) via the usage of alternative promoter and transcriptional start site (TSS) and is transcriptionally upregulated via ATM/p53 and JNK/AP1 signaling pathways triggered by DNA double-strand breaks [31]. BLM-s is transiently expressed in the postmitotic immature neurons to promote developmental apoptosis during embryonic cortical histogenesis [31]. While BLM-s is also expressed in multiple brain regions of adult mice, including the hippocampus, the physiological role of BLM-s in the adult brain is totally unknown. To better understand the physiological role of *Blm-s* gene in vivo, we generated a *Blm-s*-knockout (*Blm-s*^*−/−*^*)* mouse line. Here, we reported our macro-, meso-, and micro-scale analysis of the *Blm-s*^*−/−*^ mice to uncover the link of a proapoptotic molecule- BLM-s with anxiety and depression.

## Materials and methods

### Animals

**Fig. 1:**
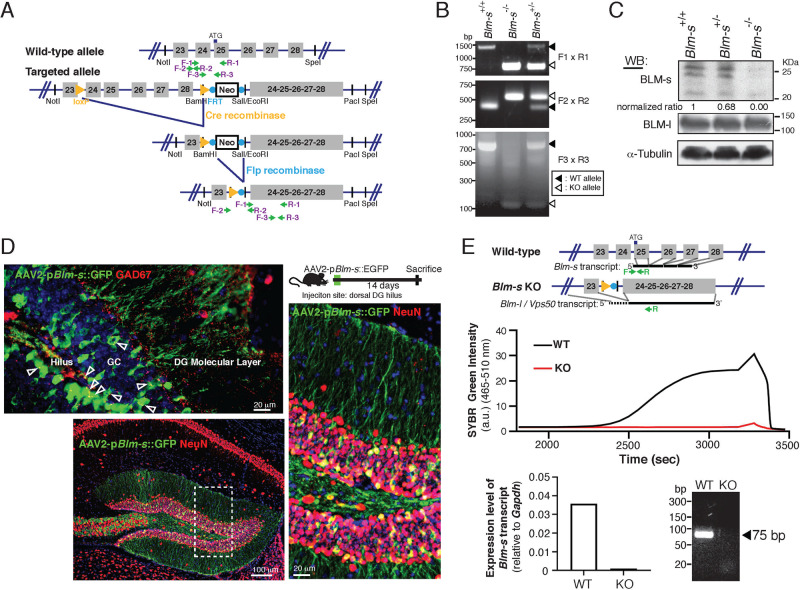
Generation of *Blm-s*-knockout mice. **A** Schematic diagram of mouse *Blm-l*/*Blm-s* genomic structure, of which *Blm-s* gene is deleted by homologous recombination and replaced with a recombinant cDNA containing exons 24–28 to preserve the *Blm-l* gene expression in the targeted allele. **B** Genotyping results of wild-type (WT, *Blm-s*^*+/+*^), heterozygotic (*Blm-s*^*+/−*^) and homozygotic *Blm-s*-knockout (KO, *Blm-s*^*−/−*^) mice. The PCR primers used are indicated in **A**. **C** Immunoblot analysis of BLM-s and BLM-l in the whole brain lysates from 60-day-old adult *Blm-s*^*+/+*^, *Blm-s*^*+/−*^, and *Blm-s*^*−/−*^ mice. α-tubulin is a loading control. **D** Schematic diagram and representative images of expression of AAV virus carrying EGFP driven by *Blm-s-*specific promoter 14 days after injection into dorsal DG of 60-old-day male wild-type mice. Note that EGFP signals colocalize with GCs (NeuN+), but not with interneurons (GAD67+). DG dentate gyrus, GC hippocampal DG granule cells. **E** Schematic diagram of a PCR primer set that specifically recognizes *Blm-s m*RNA, but not *Blm-l/Vps50* mRNA. The forward primer (F) is derived from the 5’ untranslated region of *Blm-s* transcript located in the 24th intron, which is spliced off in the *Blm-l/Vps50* transcript. Middle panel shows quantitative SYBR Green-labeled RT-PCR result amplified by this specific primer set of the hippocampal RNA derived from WT and KO mice, respectively. a.u. arbitrary unit. Lower panel reveals the expression level of *Blm-s* relative to the house-keeping gene *Gapdh* (left) and gel electrophoresis of the RT-PCR product from the hippocampi of each genotype (right).

All experiments with mice were performed in accordance with the guidelines for the Care and Use of Laboratory Animals and were approved by the National Taiwan University College of Medicine and the College of Public Health Institutional Animal Care and Use Committee (NTUMC IACUC# 20170358). *Blm-s* knockout mice were generated from the Transgenic Mouse Models Core (TMMC in NTUMC, Taiwan) using C57BL/6 J mice (purchased from the Jackson Laboratory). To specifically delete the *Blm-s* genome, we replaced the whole *Blm-s* genomic sequence, including the promoter region, (i.e., the 25–28th coding exons and associated introns of the *Blm-l* genome) with a Neo gene cassette flanked by Flippase recognition sequence followed by an engineered exon composed of the fused 24–28th exons of the *Blm-l* gene plus IRES-eGFP. As demonstrated in Fig. 1, we successfully generated *Blm-s*-specific knockout mice. However, while eGFP proteins could be detected by Western blot in heterozygotic or homozygotic *Blm-s* mutants, we were, for an unknown reason, unable to detect any fluorescent signals in any tissue sections from mutant mice.

### Genotyping, RNA extraction, quantitative RT-PCR primers, immunohistochemistry, stereotaxic injection, Golgi‑Cox impregnation, cell culture, and TUNEL assay

All experimental procedures for these assays, as well as PCR primers and antibodies used, are described in Supplementary Information.

### Behavioral analysis

All behavioral training and testing were carried out in accordance with the behavioral schemes described [36]. Animals were tested during the light cycle and were transferred to the behavioral testing room for 1 h before the start of the experiments. The sample size in each group was approximately comparable whenever possible. Provided in the Supplementary Information are the protocols and analytic methods for FST, TST, OFT, MWM and reversed MWM, EPM, Sucrose preference test, Marble burying test, NSF, Hot/cold plate test, Rotarod performance test, NOR test, and OPR test.

### Electrophysiology

Male mice with ages from 8 to 10 weeks were used. The hippocampal slices were prepared as described previously [37]. During experiments, slices were placed in a recording chamber and superfused with oxygenated artificial cerebrospinal fluid (aCSF) containing the following (in mM): 125 NaCl, 25 NaHCO_3_, 25 glucose, 2.5 KCl, 2 CaCl_2_, 1.25 NaH_2_PO_4_, and 1 MgCl_2_ at 23 ± 2 °C using a temperature controller (TMP5b, Supertech Instruments, UK). The mature GCs located at the outer 1/3 of the granule cell layer (GCL) were visually selected under an infrared and differential interference contrast (IR-DIC) microscope (BX51WI, Olympus, Japan) equipped with an infrared-sensitive charge-coupled device camera (C7500–50, Hamamatsu, Japan). Whole-cell recordings were performed with a digitizer (Digidata 1440 A, Molecular Devices, USA)-equipped amplifier (Axopatch 200B or 700B, Molecular Devices, USA). Recording electrodes were filled with the low Cl^-^ internal solution containing the following (in mM): 136.8 K-gluconate, 10 HEPES, 7.2 KCl, 7 Na2-phosphocreatine, 4 MgATP, 0.5 Na_3_GTP, 0.2 EGTA, and 0.4 % (w/v) biocytin (pH 7.3 adjusted with KOH). For all the recordings, the pipette capacitance was fully compensated, and series resistance was compensated to ~80% (Bandwidth: 1–2 kHz) in the current-clamp configuration. Signals were low-pass filtered at 2 kHz using a 4-pole Bessel filter and sampled at 10 kHz. Pulse sequences were generated by pClamp 10.2 or 10.3 (Molecular Devices, San Jose, CA, USA). Electrophysiological data analysis is described in the Supplementary Information.

### MRI experiment and data analysis

See Supplementary Information for more information. Briefly, a total of 14 WT mice and 12 *Blm-s*^*−/−*^ mice were scanned on a 7 T MRI system (Bruker Biospec, Germany). Initial anesthesia was carried out using 4% isoflurane mixed with oxygen, followed by 0.5 mL dexmedetomidine (Orion Corporation, Espoo, Finland) with a dose of 0.2 mg/kg body weight through i.p. injection. Isoflurane was switched off 10 min before the resting-state functional MRI (rs-fMRI) scan to minimize the impact of isoflurane on rs-fMRI data and was turned on before the diffusion tensor imaging (DTI) scan to minimize unwanted head motion during DTI acquisition. Both global and local shim procedures were performed to improve the field homogeneity. T2-weighted structural images were acquired using a fast-spin-echo sequence. For rs-fMRI, 1 or 2 sessions of gradient-echo echo planar imaging (EPI) with 250 repetitions were acquired using the identical geometric position as used for anatomical images. For DTI scan, 5 unweighted diffusion images (b0) and 30 diffusion-weighted images with b = 1000 s/mm^2^ were acquired. Image pre-processing and analysis were performed by using AFNI toolbox (http://afni.nimh.nih.gov), region of interest (ROI) was manually depicted according to Allen mouse brain atlas (https://mouse.brain-map.org), and resting-state connectivity among these areas was assessed by calculating the correlation coefficients between every 2 regions [19], and the amplitude of low-frequency fluctuations (ALFF) [38] was evaluated to characterize the rs-fMRI activity in WT and KO mice (see Supplementary Information for more explanations).

### Human UK Biobank data analysis

The subjects in the UK Biobank were recruited from 22 assessment centers across the United Kingdom, and additional data were collected via online questionnaires. Biological samples were also collected for genotyping. For detailed information on participants, please see https://www.ukbiobank.ac.uk/. The genetic data released contained ~96 million variants using either Affymetrix UK BiLEVE Axiom or the Affymetrix UK Biobank Axiom arrays (Santa Clara, CA, USA). Imputation was conducted centrally by UKB using two different reference panels (The Haplotype Reference Consortium (HRC) panel and UK10K + 1000 Genomes panel). We conducted standard quality control procedures for genetic variants and individuals, respectively.

We used all variables related to five domains, i.e., mood, anxiety, sleep, suicide, and psychosocial and well-being, which were collected at the assessment centers and online questionnaires. For instance, the mood domain contained 82 mood-related traits, which could be subdivided into 54 depressive traits and 28 manic traits. In total, there were 37 anxiety-related traits in the anxiety domain. Before testing genetic association, we evaluated population stratification by using principal component analysis. We performed logistic and linear regression analyses with the additive genetic model, and all models were adjusted for sex, age, and the top 10 principal components. We retained SNPs with association *p* < 1 × 10^−4^ and r^2^ < 0.5 within the gene region for significant signals.

### Statistics and reproducibility

Statistical analyses were performed using SigmaPlot 12 (Systat software). The results from behavioral tests were assayed using one-way, two-way, or two-way repeated measures ANOVAs followed by Tukey post hoc tests. The results from the quantification of TUNEL-positive cells were tested using two-tailed unpaired *t*-test. The sample size was chosen based on the literature in general without arbitrary exclusion of data points from statistical analyses. All experiments were all independently repeated with similar observations and represented either as mean ± s.e.m. or as mean ± s.d.

## Results

### Generation of *Blm-s*-knockout mice

To investigate the function of BLM-s in vivo, we generated *Blm-s*-knockout (*Blm-s*^−/−^) mice. Because *Blm-s* transcript is an isoform of the *Blm-l* gene and is transcribed via the usage of alternative promoter and TSS located at the 24th intron of the *Blm-l* genome [31], we generated a *Blm-s*-specific knockout by removing the *Blm-s* genome but preserving the transcription of *Blm-l* gene (Fig. 1A and see Materials and Methods section). RT-PCR analysis of mutant genome showed successful gene targeting (Fig. 1B). Western blot analysis of brain homogenates demonstrated null expression of BLM-s in *Blm-s*^−/−^ mutants and a gene dose-dependent loss of BLM-s protein in *Blm-s*^+/−^ heterozygotes (Fig. 1C). *Blm-l* expression was largely preserved in *Blm-s*^−/−^ mutants. Both *Blm-s*^−/−^ and *Blm-s*^+/−^ animals were born at the expected Mendelian frequency. They were morphologically normal, fertile, and viable, with life expectancy similar to wild-type (WT) mice (Supplementary Fig. S1A). The *Blm-s*^-/-^ mice had histologically normal appearing brains and internal organs as examined by H&E stain (Supplementary Fig. S1B–E). The overall brain size and weight were similar between *Blm-s*^−/−^ and WT littermates (Supplementary Fig. S1D, E).

We have previously reported that BLM-s modulate the survival of immature postmitotic migratory neurons in the developing cortex under the stress of DNA double-strand breaks. RNAi-mediated knockdown of BLM-s in murine embryonic brains protects immature neurons from irradiation-induced apoptosis [31]. Accordingly, we assessed whether *Blm-s*^−/−^ embryonic cortex is more resistant to apoptosis provoked by γ-irradiation. In line with our previous observation, the apoptosis rate of the developing neocortex evaluated by TUNEL assay was reduced in the *Blm-s*^−/−^ compared with the WT (Supplementary Fig. S1H). However, histological analysis at postnatal day 9 (P9) *Blm-s*^−/−^ cortex revealed proper expression of neocortical layer-specific markers, normal cortical layer thickness, and gray layer index compatible with age-matched WT littermates (Supplementary Fig. S1F, G), suggesting a redundant role of BLM-s in controlling postnatal neuronal numbers and brain size.

### Expression of *Blm-s transcript* in the adult hippocampus

To understand the physiological functions of *Blm-s*, we next surveyed the expression of *Blm-s* in adult brain tissue. One caveat was that the anti-BLM-s-immunohistochemistry could not precisely distinguish BLM-s-expressing cells from those expressing other ~20 isoforms of BLM, including BLM-l (i.e., mVPS50). Given that *Blm-s* mRNA is differentially transcribed by alternative usage of promoter and TSS [31], we used recombinant adeno-associated virus (rAVV) containing *Blm-s*-specific promoter to drive GFP expression in endogenous BLM-s-expressing cells. We found that GFP driven by *Blm-s* promoter expressed in the granule cells (GCs) of dentate gyrus (DG) (Fig. 1D and Supplementary Fig. S2A). Besides, GCs also showed general immunoreactivity to yet-to-be-defined isoforms of BLM-l/VPS50 that can be detected by an anti-BLM-l specific antibody (Supplementary Fig. S2B). We also designed a pair of primers specifically targeted to the 5’ untranslated region of *Blm-s* mRNA transcript that can differentiate *Blm-s* mRNA from *Blm-l* transcript. As shown, this primer set successfully recognized *Blm-s* transcript in WT hippocampus but not in *Blm-s*^−/−^ mice (Fig. 1E). We, therefore, concluded that *Blm-s* was expressed in the DG of the adult hippocampus, and the expression of *Blm-s* was depleted in the *Blm-s*^−/−^ hippocampus.

### *Blm-s*-knockout mice exhibit normal learning and memory behavior

**Fig. 2:**
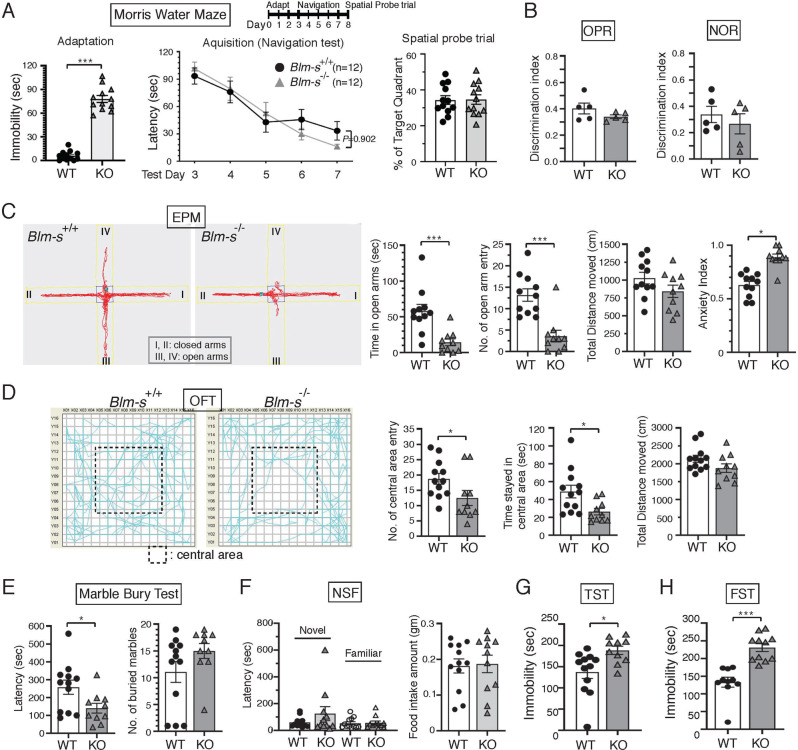
Effects of BLM-s deficiency on memory task and emotional control. **A** Experimental timeline for Morris water maze (MWM) test. Bar graphs show immobility duration of the mice in adaptation period (left) and the percentage of mice crossing the target quadrant during spatial probe trial (right). Line graph is for the escape latency of the mice to search for hidden platform during navigation stage. *Blm-s*^*−/−*^ mice (KO) showed prolonged immobility during adaptation period in the MWM test (***P < 0.001, Two-tailed Student’s *t*-tests). However, both groups showed significant negative regression learning curve as the block effect during navigation test (Two-way repeated measures ANOVA with Holm-Sidak post hoc comparison) and spent more time in the target quadrant than in the other three quadrants in spatial probe trial (Two-tailed Student’s *t*-tests). Errors are shown as mean ± s.e.m. *n* = 12 and 12 mice for WT and KO, respectively (see Supplementary Fig. S3A for representative swimming path of KO and WT mice during the MWM arena). **B** NOR (novel-object recognition) and OPR (object-place recognition) short-term memory (performed 90 min after encoding phase) were not affected by BLM-s deficiency. (See Supplementary Fig. S4H–J for graphic depict of both task arena and for discrimination ratio during the retrieval phase of long-term memory test performed at 7-day after training/encoding). **C**–**F** Evaluation of anxiety-like behaviors by elevated plus maze (EPM) (**C**), open field test (OFT) (**D**), marble burying test (**E**), and stress-induced novelty suppressed feeding test (NSF) (**F**). KO mice spent less time and number of entry in the elevated open arms during EPM test (**C**) and in the center during the open field assay (**D**) (*n* = 10 KO and 11 WT mice in EPM; *n* = 10 KO and 12 WT mice in OFT; 3 independent experiments. ***P < 0.001, *P < 0.05, Two-tailed Student’s *t*-test. Graphs indicate mean ± s.e.m.). Marble burying index (30 min) by the latency time for starting to dig marbles is much lower in KO mice (*n* = 10 KO and 12 WT mice; *P < 0.05, Mann–Whitney rank sum test), although differences in the total number of buried marbles were not seen (**E**). KO mice after fasting tended to hesitate in the first bite of food under novel environment in spite of similar food intake amount (*n* = 11 KO and 12 WT mice; Two-tailed Student’s *t*-test) (**F**). **G**, **H** Assessment of depression-like behavior by tail-suspension test (TST) (**G**) and forced swimming test (FST) (**H**). KO mice expressed helpless immobility in both tests. Data are shown with errors as mean ± s.e.m. (*n* = 10-11 KO and 10–12 WT mice; Two-tailed Student’s *t*-test).

The hippocampus is essential for learning, memory, and emotional control. To understand the in vivo functions of BLM-s, we performed behavior assays on *Blm-s*^*−/−*^ mice. First, we tested hippocampus-dependent spatial learning and memory by subjecting the *Blm-s*^*−/−*^ mice to the Morris water maze (MWM) with age-matched WT mice as controls. Both *Blm-s*^*−/−*^ and WT mice gradually learned relatively direct paths to the goal over the course of days, although the *Blm-s*^*−/−*^ showed significant immobility during the initial adaptation period (Fig. 2A and Supplementary Fig. S3A). At retrieval, both *Blm-s*^*−/−*^ and WT mice spent similar more time in the target quadrant (Fig. 2A). To rule out locomotion or sensory differences, we also tested the responses of *Blm-s*^*−/−*^ mice on the rotarod, cold plate, and hot plate, which did not exhibit any differences from the WT mice (Supplementary Fig. S3B–D).

We next examined the performance of *Blm-s*^*−/−*^ and WT mice in the reversal learning in MWM to exclude any subtle hippocampal-mediated spatial memory defects that might be caused by the loss of BLM-s. Both groups successfully learned to navigate to the hidden platform during the first three days (the first acquisition phase), with no significant difference in path length, swim speed, and latency time to the hidden platform (Supplementary Fig. S4A–D). In the first probe trial on day 4 when the platform had been moved to the opposite quadrant, *Blm-s*^*−/−*^ mice, like WT controls, spent significantly more time in the previous goal quadrant than in other quadrants, suggesting successful spatial learning in both groups (Supplementary Fig. S4E, F). After reversal learning for two days, the probe trial showed that both groups had adapted to the changed situation to develop an appropriate new spatial preference (Supplementary Fig. S4G). Although qualitative analysis of the searching strategies used in WT and *Blm-s*^*−/−*^ mice was not performed, these results together suggest that *Blm-s*^*−/−*^ mice show no obvious impairments in cognitive flexibility for hippocampal-mediated spatial learning and memory.

We also performed object-place recognition (OPR) and novel-object recognition (NOR) memory tasks on *Blm-s*^*−/−*^ mice (Supplementary Fig. S4H). OPR relies on the mouse’s intrinsic preference for novelty without additional external reinforcement, so is a simple and effective alternative for measuring hippocampal spatial learning. Consistent with the MWM results, the OPR memory did not differ between *Blm-s*^*−/−*^ and WT mice (Fig. 2B and Supplementary Fig. S4I). On the other hand, the NOR memory relies on an intact perirhinal cortex with no requirement of normal hippocampal function and is considered a non-hippocampus-dependent memory. After task encoding, both *Blm-s*^*−/−*^ and WT mice showed similar significant NOR memory at the recent (1.5 h) and remote memory (7 d) tests (Fig. 2B and Supplementary Fig. S4J). Putting it all together, *Blm-s*^*−/−*^ mice do not show impairments in spatial learning and memory in general.

### *Blm-s*-knockout mice reveal depression- and anxiety-related behaviors

The hippocampus is also functionally involved in mood control. Mood disorders such as depression or anxiety are causally related to hippocampal dysfunction. The elevated plus maze (EPM), open field test [39], marble burying test (MBT), and novelty suppressed feeding test (NSF) were used to assess whether the *Blm-s*^*−/−*^ mice had defects in anxiety-like behavior. There were significant differences in all the parameters of the EPM in the *Blm-s*^*−/−*^ mice. The mutant mice significantly reduced open space exploration and spent more time in closed arms (Fig. 2C). In the OFT, the *Blm-s*^*−/−*^ mice made fewer entries into the central zone and spent less time to stay the central area (Fig. 2D). Besides, *Blm-s*^*−/−*^ mice tended to bury more marbles and significantly decreased latency of digging performance during the MBT (Fig. 2E). In the NSF test, the food-deprived *Blm-s*^*−/−*^ mice exhibited a slower trend to start chewing in the novel environment. However, when placed in a familiar environment, there was no difference in feeding latency between *Blm-s*^*−/−*^ mice and WT mice (Fig. 2F). The total food intake in *Blm-s*^*−/−*^ mice was not different from that of the WT (Fig. 2F), suggesting that the behavioral difference in NSF test was not due to poor appetite. These results indicate that *Blm-s*^*−/−*^ mice exhibit anxiety-like behavior. We next probed depression-related traits in the *Blm-s*^*−/−*^ mice using the tail-suspension test (TST) and forced swim test (FST), in which mice show epochs of immobility that are thought to reflect behavioral despair intersected by periods of active escape movement. *Blm-s*^*−/−*^ mice showed longer periods of immobility in both tests (Fig. 2G, H). Together, these behavior tests demonstrate that BLM-s deficiency confers a genetic predisposition in adult mice with increased susceptibility to anxiety- and depression-like behaviors.

### Abnormal ventral hippocampal connectivity in *Blm-s* knockout mice via imaging study

**Fig. 3:**
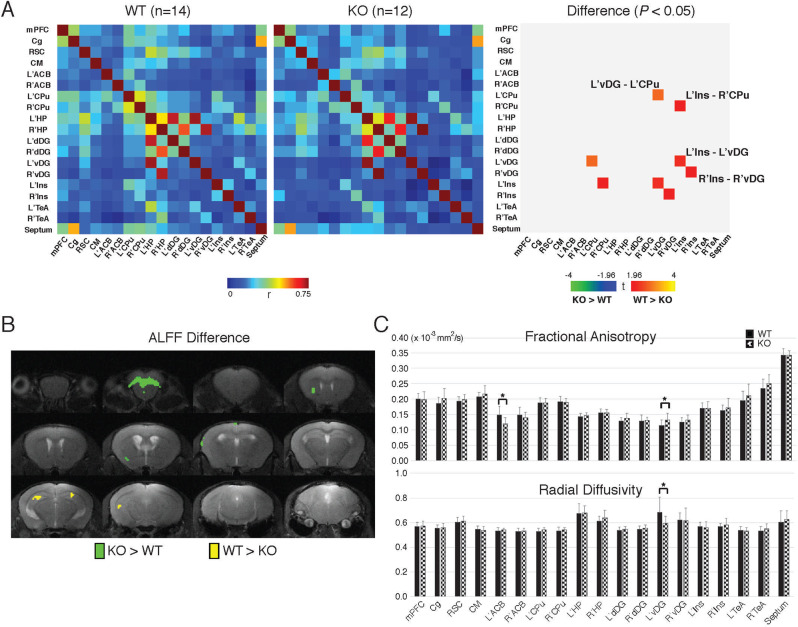
Reduced ventral DG-caudate nucleus/putmen and ventral DG-insula connectivity in *Blm-s*^−/−^ mice. **A** State brain connectivity was accessed by calculating the correlation matrix among 11 ROIs. The MRI images were preprocessed via AFNI toolbox and the signal time course in each ROI was obtained, where the 11 signal time courses were then compared to each other to generate the correlation matrix in each mouse. ACB nucleus accumbens, Cg cingulate gyrus, CM central medial thalamic nucleus, CPu caudate nucleus/putmen, dDG dorsal DG, vDG ventral DG, HP hippocampus, Ins insular cortex, mPFC medial prefrontal cortex, RSC retrosplenial cortex, TeA temporal association cortex (see Supplementary Fig. S5A for graphic illustration of ROI). *n* = 12 *Blm-s*^−/−^ (KO) and 14 WT mice, Two-sample *t*-test (*P* < 0.05 as significant). **B** State activities were evaluated and compared by calculating the amplitude of low-frequency fluctuations (ALFF) in a voxel-wise manner. Higher activity of ALFF was shown in the hippocampus of KO mice. **C** The DTI parameter comparison in various brain ROIs of KO and age-matched WT mice is shown by bar graph, indicating altered fractional anisotropy (FA) and axial diffusivity (AD) in the hippocampus of KO mice. *n* = 12 KO and 14 WT mice, *P < 0.005, Two-sample *t*-test.

The anxiety and depression-like behaviors found in adult *Blm-s*^*−/−*^ mice are likely to be caused by a disrupted neural network. Brain neuroimaging such as rs-fMRI and DTI can be used to identify the brain-wide activity patterns and connectivity maps that underlie behaviors. *Blm-s*^*−/−*^ mice (*n* = 12) and age-matched WT mice (*n* = 14) were scanned under dexmedetomidine anesthesia (0.2 mg/kg) on a 7 T animal MRI system. Analysis of preprocessed rs-fMRI images among ROIs involved in mood control (Supplementary Fig. S5A) indicated that compared to WT mice, *Blm-s*^*−/−*^ mice showed reduced connectivity in the following pairs of regions: left ventral dentate gyrus (vDG)—left caudate putamen (CPu), left insula—right CPu, left insula—left vDG, and right insula—right vDG (Fig. 3A). These data suggest that *Blm-s*^−/−^ mice had reduced connectivity centering in the hippocampus, particular in the vDG. Note that the MRI results did not show significant connectivity change in the dorsal DG (dDG) of *Blm-s*^*−/−*^ mice when compared to that of the WT mice. Besides, the hippocampal volume of *Blm-s*^*−/−*^ mice estimated by MRI was not different from that of WT (Supplementary Fig. S5B). The amplitude of low-frequency fluctuation (ALFF) was also evaluated to assess regional state activity. In the *Blm-s*^*−/−*^ mice, reduced ALFF was found in the bilateral dentate gyrus, while increased ALFF was found in the orbital cortex (Fig. 3B). The DTI analysis also revealed elevated fractional anisotropy (FA) and reduced radial diffusivity (RD) in the vDG of *Blm-s*^*−/−*^ mice (Fig. 3C), suggesting either increased fiber tract density or reduced cell number in these brain regions. Our MRI findings suggest that microstructures and local state activity in the hippocampal area, particularly in the vDG, are altered in *Blm-s*^*−/−*^ mice.

### Morphological and electrophysiological changes are observed in the dentate granule cells of the *Blm-s*-knockout mice

**Fig. 4:**
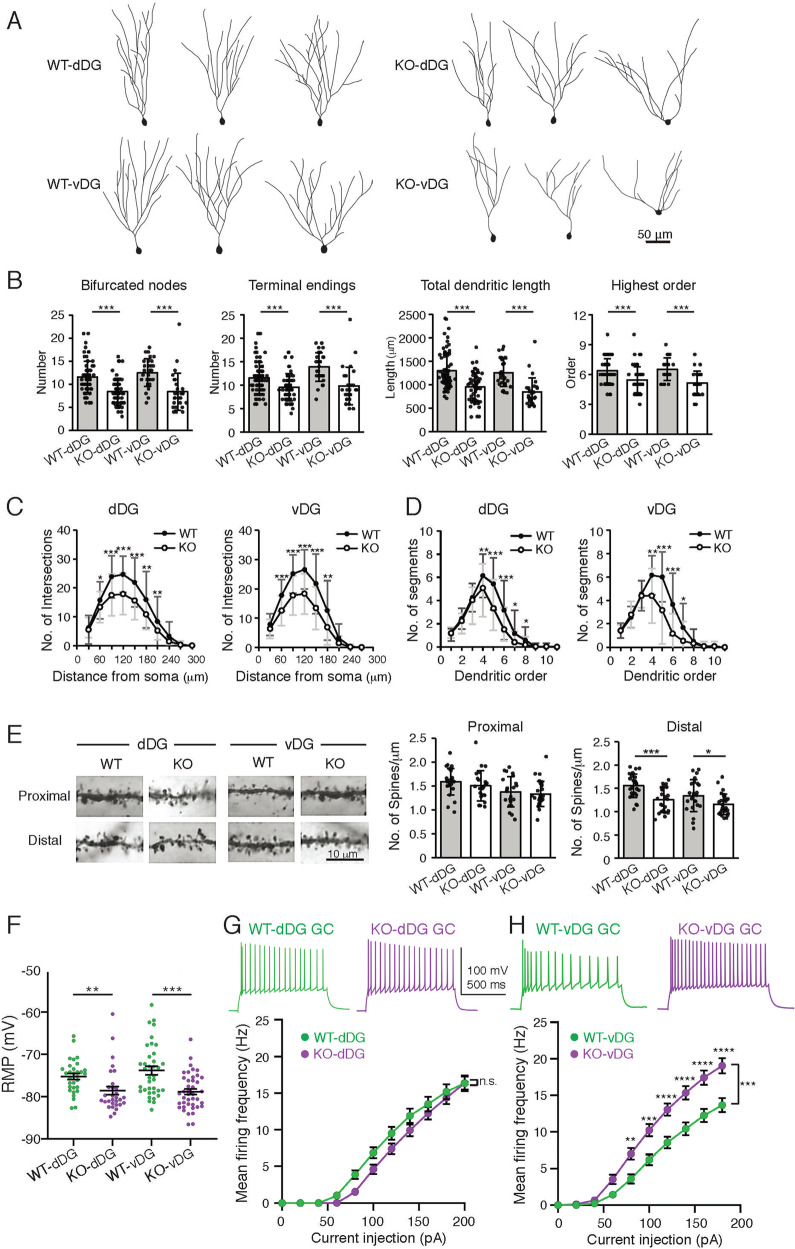
Morphological and electrophysiological changes of dentate granule cells in *Blm-s*^*−/−*^ mice. **A** Examples of reconstructed granule cells in dorsal(d)- and ventral(v)-dentate gyrus (DG) of adult *Blm-s*^*+/+*^*(*WT) and *Blm-s*^*−/−*^(KO) mice analyzed by Neurolucida (version 5.1.2) software of Golgi-stained hippocampal slices. **B** Morphological parameters of dendritic patterns of DG granule cells analyzed by Neurolucida software including dendritic tree bifurcation number, terminal numbers, total dendritic length and dendritic branch orders. **C**, **D** Dendritic complexity assessed by Sholl analysis shown by number of intersections along the distance from the soma (**C**) and the number of segments for each dendritic order (**D**). **E** Representative image of dendritic spines (left) and the calculated density of dendritic spines (right). All Data in **B**–**E** are presented as mean ± s.e.m. *n* ≧ 25 neurons from three mice for each group. *P < 0.05, **P < 0.01, ***P < 0.001, two-tailed unpaired Student *t*-test. **F** Summary of the resting membrane potential (RMP) of *Blm-s*^*−/−*^ (KO) (magenta)- and WT (green)- GCs from dorsal DG (labeled as KO-dDG: *n* = 31, WT-dDG: *n* = 31, respectively) and ventral DG (labeled as KO-vDG: *n* = 41, WT-vDG: *n* = 37, respectively). **P < 0.01, ***P ≤ 0.001, unpaired *t*-test with Welch’s correction. **G**, **H** Representative voltage responses of GCs to 200 pA current pulse injections from dorsal DG (**G**) or ventral DG (**H**) of WT and KO mice. Note that membrane potential before the pulse injection was ~−80 mV for both genotypes. The plot summarizes the mean firing frequency versus current injection in dorsal DG GCs (**G**) or ventral DG GCs (**H**) from WT and KO mice. n.s. nonsignificant (*P* = 0.1374 in (**G**)), **P < 0.01, ***P < 0.001, ****P < 0.0001, α = 0.05, two-way ANOVA with the Bonferroni post hoc test. Errors in **F**–**H** are shown as mean ± s.e.m.

The behavioral impairments and imaging abnormalities in *Blm-s*^*−/−*^ mice reflect alterations in neuronal circuitry of the hippocampal DG. Since BLM-s is expressed in the DG GCs, we analyzed the morphological and electrophysiological changes of the GCs in the *Blm-s*^*−/−*^ DG. Because the behavior and imaging studies suggested specific involvement of vDG, the morphological characteristics of GCs in the vDG were examined separately from dDG by Golgi-Cox stain (Fig. 4A). The dendritic morphology, length complexity, and segmentations of the GCs were significantly decreased in both vDG and dDG of the *Blm-s*^*−/−*^ mice (Fig. 4B–D). The spine density was also significantly reduced on the distal dendrites of the *Blm-s*^*−/−*^ GCs (Fig. 4E). These morphological changes in vivo might be specific to *Blm-s*^*−/−*^ DG GCs, because the embryonic cortical neurons cultured from *Blm-s*^*−/−*^ and WT mice in vitro did not show differences in dendritic morphology (Supplementary Fig. S6A). Given the abnormality of dendritic morphology and spine density in the *Blm-s*^*−/−*^ DG GCs, we examined the ultrastructure of the excitatory (asymmetric) synaptic contacts in the DG by transmission electron microscopy and found that the PSD (postsynaptic density) length and area were altered in the asymmetric synapses of *Blm-s*^*−/−*^ DG GCs (Supplementary Fig. S6B, C).

To assess the functional changes of mutant DG GCs, we performed electrophysiological analysis on the hippocampal slices from the *Blm-s*^*−/−*^ and age-matched WT mice. The intrinsic properties of GCs, including resting membrane potential (RMP) and spike properties, were compared between WT and *Blm-s*^*−/−*^ DG along the dorsal-ventral axis. *Blm-s*^*−/−*^ GCs from both dorsal and ventral DG (labeled as KO-dDG and KO-vDG) exhibited more hyperpolarized RMP compared with those from WT-dDG and WT-vDG, respectively (Fig. 4F). Interestingly, *Blm-s*^*−/−*^ GCs from vDG generated more action potentials (APs) in response to depolarizing currents greater than 80 pA (Fig. 4H), while *Blm-s*^*−/−*^ GCs from dDG generated similar firing frequency with the WT-dorsal DG GCs (Fig. 4G). Analysis of rheobase revealed that the minimal current injections required for AP generation in ventral *Blm-s*^*−/−*^ DG GCs were less than those in ventral WT DG GCs, while there was no difference in rheobase between *Blm-s*^*−/−*^- and WT-dorsal DG GCs (Supplementary Fig. S7A). Nevertheless, analysis of the input resistance (R_in_), AP threshold, and AP peak amplitude evoked by rheobase current injection revealed no significant difference between *Blm-s*^*−/−*^- and WT-GCs either from dorsal- or from ventral DG (Supplementary Fig. S7B–D). Together these results indicate that the mature GCs from *Blm-s*^*−/−*^ DG, particularly in the ventral DG, show significant morphological, synaptic, and functional changes that might contribute to altered DG network function in the *Blm-s*^*−/−*^ mice.

### Human *Blm* homologue genetic variants are associated with an increased risk for depression

**Fig. 5:**
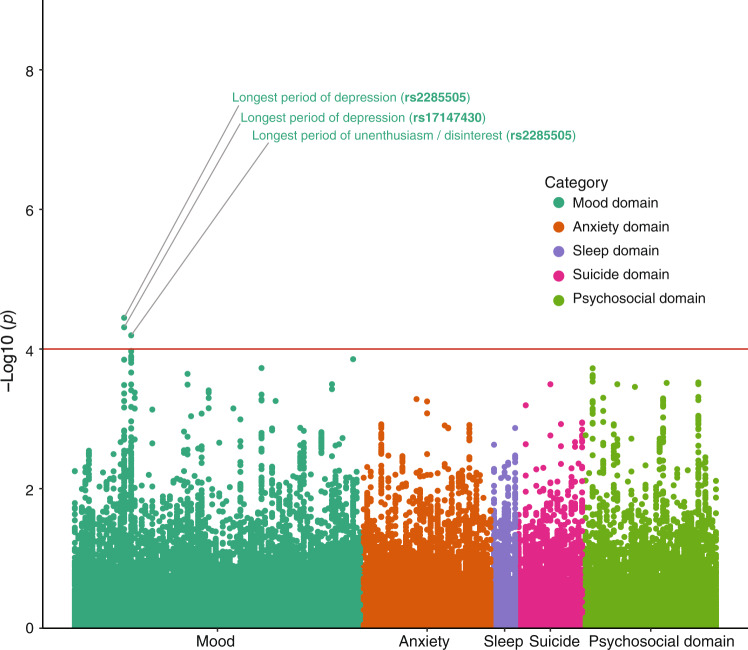
Manhattan plot of the probability for *VPS50* variants in association with five domains related to major depression. Two hundred seventy-three SNP (single nucleotide polymorphism) variants in human VPS50 gene (human homolog of mouse *Blm*) derived from UK Biobank (https://www.ukbiobank.ac.uk/) were analyzed for five domains (mood, anxiety, sleep, suicide, and psychosocial factors) related to major depression that containing 183 traits in total. SNP variants with association *p* < 1 × 10^−4^ and r^2^ < 0.5 were considered as significant signals. Two variants rs2285505 and rs17147430 are significantly associated with mood domain.

To explore whether BLM-s homologue in humans is functionally associated with mood control, we turned to search for SNP (single nucleotide polymorphism) variants of the human *Blm* (*VPS50)* gene in correlation with any depression-related traits from public assessable UK Biobank data (https://biobank.ndph.ox.ac.uk/showcase/). The genomic locations were based on the human genome GRCh37 assembly and defined from 5’ to 3’ region with an extra 50 kb. A total of 273 variants in *VPS50* gene were retained for analysis. We analyzed 5 domains (mood, anxiety, sleep, suicide, and psychosocial factors) containing 183 traits in total. After quality control, we used 425,214 individuals with European ethnic backgrounds in the UK Biobank to perform an association analysis. We performed logistic and linear regression models with additive genetic modes for binary continuous traits, respectively. All models were adjusted by sex, age, and the top 10 principal components. Two variants in *VPS50* gene were significantly associated, and both associations were only in the mood domain but not in the other four domains. The first one, rs2285505, is located at intron 27 of *VPS50* gene, which is equivalent to intron 3 of human homolog of murine *Blm-s*. It was significantly associated with two specific traits, i.e., longest period of depression (*p*-value = 3.6 × 10^−5^) and longest period of unenthusiasm/disinterest (*p*-value = 6.4 × 10^−5^). The second one, rs17147430, is located at intron 9 of *VPS50* gene. It was also associated with the longest period of depression (*p*-value = 4.9 × 10^−5^) (Fig. 5). These human data, therefore, suggest a potential link between *BLM-s/VPS50* gene variants and human depression traits.

## Discussion

BLM-s is a BCL-2 family protein that has been shown to promote apoptosis of the migrating immature neurons during murine cortical development [31]. However, the *Blm-s*^*−/−*^ mice do not show any obvious developmental defects in the brain, suggesting that either BLM-s acts redundantly with other BCL-2 family members or that multiple strategies are exploited to compensate for the loss of BLM-s for normal development of brain cortices. It has been reported that deficiency of BLM-l (mVPS50), an isoform of BLM-s, in mice causes a cognitive defect, which is attributed to dysfunctional dense core formation in neurons [32]. By contrast, we show that the adult *Blm-s*^*−/−*^ mice exhibit anxiety- and depression-like behaviors while their ability for spatial memory and learning remain intact. Mesoscale analysis of *Blm-s*^*−/−*^ mice via rs-fMRI further suggests that the emotional disorders in the *Blm-s*^*−/−*^ mice could be related to a defect in the connectivity of vDG, which is critical for emotional control and is functionally segregated from the dDG that controls spatial learning and memory [40–44]. Although the hippocampal morphology, volume, and hippocampus-to-brain weight ratio in adult *Blm-s*^*−/−*^ mice are not different from the WT, the *Blm-s*^*−/−*^ mature GCs, particularly those in ventral DG, do exhibit morphological and electrophysiological abnormalities, which could underlie the microstructural changes and altered functional connectivity in the vDG. Our results thus support the synaptogenesis hypothesis of depression, which states that dysregulated synaptogenesis leads to abnormal electrophysiology of neurons and aberrant circuit activity that underlies depressive behavior [5]. Without BLM-s, the GCs in the DG, particularly in the vDG, cannot make proper connections to the DMN core regions and the striatum, so the *Blm-s*^*−/−*^ mice exhibit anxiety and depression-like behavior.

It has been hypothesized that human major depression disorder (MDD) is a polygenetic disorder resulting from accumulative effects of multiple mutated genes with minor effects each [24, 25]. Although BLM-s or its isoforms BLM-l (VPS50) is not in the list of candidate genes and variants for major MDD studies [26, 39], our analysis of UK biobank does reveal that human *VPS50* rs2285505 and rs17147430 variants are statistically significantly associated with increased depression in mood domain. This implies that BLM-s may be one of the contributing factors to human MDD. In addition to BLM-s, other BCL-2 family members have been reported to be involved in mood disorders as exemplified by dysregulated prosurvival BCL-2 and BCL-xL in anxiety, depression, or bipolar disorders in humans [45–47]. Anxiety behavior in mice has also been observed through the deletion of the proapoptotic BAX gene in murine neural progenitor cells [48]. These findings together suggest the involvement of the BCL-2 family members in mood control and their dysregulation in association with a mood disorder.

Given that dysregulation of either anti- or proapoptotic BCL-2 members is observed in mood disorders and that the hemostasis model among the BCL-2 members operates quite well in explaining their effect on cell survival [49, 50], it is possible that mood disorders associated with proapoptotic BLM-s deficit results from unbalanced over-activity of other antiapoptotic molecules such as BCL-2 or MCL-1. However, the such possibility might be small based on the following reasons. First, deficiency of either proapoptotic or antiapoptotic BCL-2 leads to the same phenotype in mood disorder, which could not be simply explained by the homeostasis hypothesis that describes a biological effect achieved by the summation of all activities among pro- and antiapoptotic BCL-2 family members. Second, BLM-s physically and functionally antagonize antiapoptotic BCL-2 and MCL-1 [31]. Accordingly, *Blm-s*^*−/−*^ mice would be expected to exhibit increased BCL-2 activity, which would lead to an antidepressive phenotype as previously reported [45] rather than causing depression. Third, it has been hypothesized that dysfunctional apoptosis leads to neuronal loss and decreased brain volume, which underlies cognitive decline and mood dysregulation in some depressive human patients [47]. However, the brain volume is not lost in the *Blm-s*^*−/−*^ mice, arguing against such a scenario. Accordingly, BLM-s in the murine hippocampus might play a role in mood control via a nonapoptotic process.

Our extensive analyses of *Blm-s*^*−/−*^ mice do imply BLM-s’ nonapoptotic role in mood control. First, recent genetic studies indicate that dysregulated developmental profiles of voltage-gated cation channels are highly associated with psychiatric diseases [51] and that BCL-2 family member BAD indeed regulates potassium channels to control cell excitability and action potential generation [52]. Thereby, the altered intrinsic properties of *Blm-s*^*−/−*^ DG GCs might result from BLM-s-mediated changes in the number, composition, location, or dynamics of ion channels within the membrane [53]. Second, decreased neuronal dendrite branching and plasticity of the hippocampus are thought to contribute to the negative effects of stress/depression and can be reversed by antidepressants [54]. The GCs in the *Blm-s*^*−/−*^ DG also exhibit fewer dendritic branches and reduced dendritic spines, which could causally relate to depression behavior in *Blm-s*^*−/−*^ mice. BLM-s may have a direct effect on the morphogenesis during the maturation of GCs in DG. Alternatively, the morphological changes of GC dendrites in *Blm-s*^−/−^ DG might result from synaptic or cellular homeostasis in response to functional alteration related to decreased neuronal connectivity. Third, accumulative data have shown that mitochondrial impairment triggers mood disorders [55–62]. A well-known nonapoptotic role of the BCL-2 family members involves the regulations of mitochondrial dynamics, metabolism, bioenergetics, calcium homeostasis, mitochondrial UPR, and mitophagy [63, 64]. Because BLM-s is localized in the mitochondria [31], depletion of BLM-s could potentially affect mitochondrial physiology in the limbic system, which in turn causes dysfunctional mood control. Further exploration of BLM-s’ role in mitochondria would be interesting, given that drugs targeting mitochondria physiology are currently investigated as a potential pharmaceutical target for the treatment-resistant major depressive disorder [60].

## Supplementary information

Supplementary materials and methods

Supplementary Figure S1

Supplementary Figure S2

Supplementary Figure S3

Supplementary Figure S4

Supplementary Figure S5

Supplementary Figure S6

Supplementary Figure S7
