# Supplementary material for: Involvement of a BH3-only apoptosis sensitizer gene Blm-s in hippocampus-mediated mood control
Source: Transl Psychiatry. 2022 Sep 26;12:411. doi: 10.1038/s41398-022-02184-6 (PMC9512807; doi:10.1038/s41398-022-02184-6)
Supplement: Supplementary file 1 — Supplementary materials and methods [file 41398_2022_2184_MOESM1_ESM.docx]

**Supplementary Information**

**This Supplementary Information includes 1) Supplementary Material and Methods and 2) Supplementary Figures S1-S7 and Supplementary Figure Legends.**

**Involvement of a BH3-only apoptosis sensitizer gene *Blm-s* in hippocampus-mediated mood control**

Pei-Hsin Huang^1,2,#^, Tseng-Yin Yang^1,^*, Chia-Wei Yeh^3,^*, Sheng-Min Huang^4,^*, Ho-Ching Chang^5,^*, Yun-Fen Hung^5,^*, Wen-Chia Chu^1^, Kuan-Hung Cho^4^, Tzu-Pin Lu^6^, Po-Hsiu Kuo^6,7^, Li-Jen Lee^8,9^, Li-Wei Kuo^4,10^, Cheng-Chang Lien^3,11^, Hwai-Jong Cheng^5^

^1^Graduate Institute of Pathology, ^8^Graduate Institute of Anatomy and Cell Biology, ^9^Institute of Brain and Mind Sciences, and ^10^Institute of Medical Device and Imaging, College of Medicine, and ^6^Department of Public Health & Institute of Epidemiology and Preventive Medicine, College of Public Health, National Taiwan University, Taipei 100, Taiwan

^2^Department of Pathology, and ^7^Department of Psychiatry, National Taiwan University Hospital, Taipei 100, Taiwan

^3^Institute of Neuroscience, College of Life Sciences, and ^11^Brain Research Center, National Yang Ming Chiao Tung University, Taipei 112, Taiwan

^4^Institute of Biomedical Engineering and Nanomedicine, National Health Research Institutes, Miaoli 350, Taiwan

^5^Institute of Molecular Biology, Academia Sinica, Taipei 115, Taiwan

* These authors contribute equally to this work.

^#^Author for correspondence: Pei-Hsin Huang, e-mail: phhuang@ntu.edu.tw

**Supplementary Materials and Methods**

**Data reporting**. The investigators are blind to allocation of mouse genotypes during data analysis and behavior assessment.

**Animals** **and Genotyping**. All mice were crossed and housed in standard cages in a temperature controlled (22^o^C) room under diurnal condition (12h/light/dark cycle). Food and water were provided ad libitum unless otherwise noted for behavioral experiments. Mice were analyzed with the following primers to genotype progenies using PCR: F1 forward: 5’-ATA GAA TTC TGA CTG TCC TGA GAG AGT T-3’, R1 reverse: 5’-GTT CAT AAT CAA TAG CTT TAC CAG CC-3’; F2 forward: 5’-ATA GCG GCC GCA TCA GCA AGA ATG CCT TC- 3’, R2 reverse: 5’-ATA GTC GAC ACC ATG TCA ATC CTA GGA A- 3’; F3 forward: 5’-TCT TGG CTG AGC AGT TTG AG- 3’, R3 reverse: 5’-GCT TTA CCA GCC ACA ATC C- 3’. The sizes of PCR products by primers F1 x R1: targeted allele is 799 bp and wild-type allele is 1425 bp; F2 x R2: targeted allele is 600 bp and wild-type allele is 396 bp; F3 x R3: targeted allele is 151 bp and wild-type allele is 779 bp.

**RNA extraction and quantitative RT-PCR.** Total RNA was extracted from mouse brain using TRIzol reagent according to the manufacturer’s instructions (Ambion/Life technology, Carlsbad, CA, USA), followed by DNase I (Sigma-Aldrich, St. Louis, MO, USA) digestion to remove contaminating DNA. cDNA was reverse-transcribed for real-time PCR. The primers for Q-PCR: *Blm-s* forward: 5’-GTT TTT TGT TTC TGT GGA ACA TTT CAG-3’; *Blm-s* reverse: 5’-GCC ACA ATC CAG TAA ATG GGC TTG-3’; *Gapdh* forward: 5’-GGG TTC CTA TAA ATA CGG ACT GC-3’; *Gapdh* reverse: 5’-CCA TTT TGT CTA CGG GAC GA-3’. A real-time PCR assay was performed using the SYBR Green (F. Hoffmann-La Roche, Basel, Switzerland) system.

**Stereotaxic injection**. All surgeries were performed using aseptic techniques and animals were pre-operatively injected with the anesthetics/analgesics. All stereotaxic reference points were set at Bregma for the AP axis, at the midline for the ML axis, and at the surface of the brain for the DV axis. For viral infection at dentate gyrus, animals received bilateral stereotaxic infections of the indicated viruses at rates < 0.1 ml min^-1^ with the injection coordinates: AP= -0.5 mm, ML= ±2.5-3.0 mm, DV= 0.8 mm. All brain section schematics for showing virus injection site were drawn using Adobe Illustrator to closely resemble the corresponding sections in the Paxinos brain atlas.

**Immunohistochemistry**. Animals were intracardially perfused with 4% paraformaldehyde (PFA)(Sigma-Aldrich) in PBS and the brain was dissected out, post-fixed with 4% PFA overnight at 4^o^C. For cryo-sectioning, the fixed brain was cryo-protected with 30% sucrose solution, embedded in Tissue Tek OCT (Sakura Finetek, Torrance, CA, USA) and sliced either coronally or sagittally at 14 μm using a cryostat (Leica, Wetzlar, Germany). For vibratome sectioning, the brains were coronally sliced at either 50 or 100 μm with a Leica VT1000S vibratome (Leica).

Slices were permeabilized with blocking solution containing 0.3% Triton X-100 and 3% goat serum in PBS for 1 hr at room temperature and then incubated with primary antibodies (rabbit anti-BLM-l (1:1000), mouse anti-BLM-s (1:50)(1), mouse anti-GFP (8 μg/mL)(clone 3E6 #AB_221568, Invitrogen/Thermo Fisher Scientific, Eugene, OR, USA), anti-GAD67(1:200)(#MAB5406, Millipore/Merk, Temecula, CA, USA), anti-NeuN (1:500)(Millipore, #ABN91) antibodies overnight at 4^o^C, followed by fluorescently conjugated secondary antibodies (1:1000, Invitrogen) for 1 hr at room temperature with H33258 (1:1000, Invitrogen) for nuclear staining. Images were acquired using a confocal microscope (LSM800; Carl Zeiss, Oberkochen, Germany) with 20x objective lens or an epifluorescence microscope (Leica DMR) with 20x/40x objective lens. All image settings were kept constant across the same batch of experimental groups.

**TUNEL assay.** Apoptotic cells in the embryonic brain 24 hr post γ-irradiation were analyzed using the *in situ* cell death detection kit-POD (Roche) as previously described (1).

**Golgi‑Cox impregnation and morphometric analyses.** Mice were intracardially perfused with 4% PFA in phosphate buffer, pH 7.4. Whole brains were processed using FD Rapid Golgi Stain kit (NeuroTechnologies, Ellicott City, MD, USA). GCs were selected from the dorsal part (from Bregma -1.58 mm to -2.06 mm) and ventral part (the lower half of sections from Bregma -3.4 mm to -3.8 mm) of DG. The spine density on proximal dendrites was calculated from the first bifurcated node and distal spine density were counted from dendrite terminals within 50 μm. The dendrite morphology and spine density of neurons was reconstructed and analyzed with Neurolucida software (MBF Bioscience).

**Cell culture, transfection, immunofluorescence staining, and microscope image acquisition.** COS and NG108 cells (purchased from ATCC) were cultured with DMEM (Dulbecco’s modified Eagle’s medium) containing 10% fetal calf serum and 1% penicillin/streptomycin (Life Technology) at 37°C in a 5% CO_2_ incubator. Mouse primary embryonic cortical neuron culture preparation, transfection and immunofluorescence staining were performed as described (2). Immunofluorescent images of fixed mature cultured neurons were recorded with a confocal microscope (LSM900; Zeiss) equipped with a 63x 1.4 NA oil objective as a z series. The images were then projected into single images for quantification.

**Behavioral analysis.**

***Forced swimming test (FST):*** Animals were subjected to FST on day 1 and day 2. The mice were placed in a glass cylinder (30 cm in height and 20 cm in diameter) filled with 20-25 ºC water to a height of 15 cm. In the first trial, animals were immersed in water for 10 min, followed by 6 min swim 24 hr later, and then tested in the 2^nd^ swim 24 hr later. Animal behavior was videotaped, and the total immobility duration was manually counted in a double-blinded way. Immobility was defined as floating or motionless without leaning against the wall of the cylinder or any movement necessary to keep their head above the water to maintain the body balance.

***Tail-suspension test (TST):*** Mice were suspended by their tail using an adhesive tape from the tip of the tail for 6 minutes (3). Their immobility behavior was videotaped on the side and total immobility duration was analyzed. The immobility was defined as lack of all sorts of movements except those required for respiration. Additionally, any tiny movements that were confined to front legs without any involvement of hind legs were also included in the definition of immobility.

***Open field test (OFT):*** Mice underwent a 5-min exploration in the testing arena (size: 40 cm x 40 cm x 35 cm). Sessions were videotaped by the EthoVision® tracking system (Noldus Information Technology, the Netherlands) and analyzed for the total time spent in the center (center size: 20 cm x 20 cm) and the number of entry into the central area.

***Morris water maze (MWM) and reversed Morris water maze:*** The apparatus consisted of a circular white pool (20 cm deep and 100 cm in diameter), partially filled with water to 15 cm in depth, and with four highly visible cues on the wall of each quadrant. A transparent hidden Plexiglas platform was positioned 1 cm below the water’s surface. Each animal was tested four trials per day for 8 consecutive days. For each trial, mice were placed in the pool facing the wall at one of the four starting locations (east, west, south, or north) randomly. In navigation test, mice were given a maximum of 2 min to find the hidden platform and the latency to reach the hidden platform was recorded. If the platform was not found by 2 min, the animal was manually placed on the hidden platform and allowed to stay there for 10 seconds (sec). Mice were placed in a warming chamber for at least 10 min between trials. For the spatial probe trial, the hidden platform was removed, and the animal was allowed for 2-min free exploration. The percentage of the mouse crossing target quadrant was recorded.

***Elevated plus maze (EPM):*** Mice were individually placed in the central area (5 cm × 5 cm) of an elevated plus maze (75 cm high), facing one of the closed arms (5 cm wide × 30 cm long × 22.5 cm high), and were allowed free exploration for 5 min. The time spent in open arm, the number of the open arm entry, the total number of arm entry, and the total moving distance were evaluated by the EthoVision® tracking system (Noldus Information Technology). The anxiety was evaluated by the following equation: anxiety index = 1- [(time in open arm / test duration) + (number of open arm entry / total number of arm entry) / 2].

***Sucrose preference test (SPT):*** Before the test, mice underwent two successive 2-day habituations to avoid extra-bottle or sweetener-novelty bias. The mice were then given a choice between water and 2% sucrose for one week. The position of the bottle was randomly changed each day. The relative daily consumption of sucrose water was calculated as the ratio over total liquid consumption.

***Marble burying test:*** Mice were individually placed into the testing cage (28.5 cm x 17.5 cm x 12.5 cm) with wood-chip bedding (5 cm in depth) containing 20 equally distributed glass marbles arranged in five rows of four. The exploration period was 30 min and the latency time starting to dig the marbles was recorded. In addition, after the mice were removed from the cage at the end of the test, the number of marbles buried, defined as a marble covered at least 2/3 of its size by dust, was recorded.

***Novelty-suppressed feeding test (NSF):*** After 24 hr deprivation of food, the animal was put in a familiar (28.5 cm × 17.5 cm × 12.5 cm) or a novel (46 cm × 24 cm × 20 cm) environment with a single food pellet (3.0-5.0 grams) placed in the center. The feeding latency was recorded and defined as the mouse sitting on its haunches and eating the food pellet using its forepaws.

***Hot/cold plate test***: Mice were placed into a glass cylinder on the plate surface (Ugo Basile Hot/Cold Plate 35100), and the response latency was recorded. The response latency was defined as the time between placement of the mouse on the hot (55.0 ± 2.0 ºC) or cold (2.0 ± 2.0 ºC) plate and the occurrence of movements such as removal, lifting, or licking of it paws, or jumping off the surface. A cut-off time of 60 sec was used to avoid injury.

***Rotarod performance test***: A rotarod machine (Ugo Basile Rota-Rod 47600) with automatic timer and falling sensor was used. The revolutions per minute (rpm) was set at 4 rpm at the beginning and was gradually increased to a maximum of 40 rpm across 5 min, with a maximum duration of 5 min per trial. The animal was placed on the rotating rod and recorded for the latency until it fell off. The test was repeated three times consecutively and the average scores were used as a measure of locomotion ability.

***Novel-object recognition (NOR) and object-place recognition (OPR) test***: To habituate the animals, mice in the home cages were individually placed into the training square box (50 cm x 50 cm x 40 cm) without any object and allowed for 5-min free exploration period per day for three days. On day 4, the mice were trained to learn the spatial position of two identical objects (250 mL beakers flipped upside-down and facing the same direction) placed in the square box for 10 min. Mice were then tested for short-term memory after 90-min rest on the training day or for long-term memory after 7-day rest in home cage. For NOR test, one of the two beakers originally used on the training day was replaced with an Erlenmeyer flask (250 mL). For OPR test, one of the two beakers was relocated to a new position and the mice were allowed to explore for 5 min. The whole procedure was under video recording. Video analysis and the timing were conducted by an experimenter blind to the experimental conditions. Mice that did not explore more than 3 sec total for both objects during the training or testing were excluded from analysis. Discrimination index was calculated as: (time exploring the novel object/location- time exploring the familiar) / (time exploring the novel + familiar).

**Electrophysiology data analysis.** Electrophysiological data were analyzed using Clampfit 10.7 (Molecular Devices). The resting membrane potential (RMP) was recorded immediately in the current-clamp configuration after breaking-in to the cells. AP trains were generated by 1-s current injection (step increment 20 pA) with the membrane potential kept at approximately -80 mV. The R_in_ was measured by dividing the amplitude of 1-s hyperpolarizing current (-10 pA) by the elicited steady-state (the last 100 ms) voltage change. The rheobase was the minimal intensity of 1-s current injection required for AP generation. The AP properties were analyzed from the AP evoked by the rheobase current injection. The AP threshold was calculated as the membrane potential at which the first derivative exceeded 20 V/s and the peak amplitude was measured from the AP threshold (5).

**MRI experiment.** A total of 14 WT mice and 12 *Blm-s^-/-^* mice were scanned on a 7T MRI system (Bruker Biospec, Germany). Initial anesthesia was carried out using 4% isoflurane mixed with oxygen, followed by 0.5 mL dexmedetomidine (Orion Corporation, Espoo, Finland) with a dose of 0.2 mg/kg body weight through i.p. injection. After the administration of dexmedetomidine, mouse was placed on the holder with ear bar and bite bar for fixation, while 1% of isoflurane was continuously introduced. Respiratory rate was continuously monitored during the whole scanning process and body temperature was kept with warm water circulation. Isoflurane was switched off 10 min before rs-fMRI scan to minimize the impact of isoflurane on rs-fMRI data, and was turned on before DTI scan to minimize unwanted head motion during DTI acquisition. Both global and local shim procedures were performed to improve the field homogeneity. T2-weighted structural images were acquired using a fast-spin-echo sequence with the following parameters: field-of-view (FOV) = 20 mm × 20 mm, matrix size = 256×256, 12 slices with 0.8 mm slice thickness, repetition time (TR) / effective echo time (TE) = 2500/33 ms, echo train length = 8, 4 averages. For rs-fMRI, 1 or 2 sessions of gradient-echo echo planar imaging (EPI) with 250 repetitions were acquired using the identical geometric position as used for anatomical images. Scanning parameters were: matrix size = 80 × 80, TR/TE = 2000/15 ms, total scan time = 8.3 min for each session. The obtained spatial resolution was 0.25 mm × 0.25 mm × 0.8 mm. For DTI scan, 5 unweighted diffusion images (b0) and 30 diffusion-weighted images with b=1000 s/mm^2^ were acquired. The scan parameters were matrix size = 128 × 128, TR/TE = 3750/31.2 ms, repetition = 4. These four repetitions were averaged out for DTI reconstruction. After MRI scanning, antidote atipamezole was administered to reverse the sedative effect.

**MRI data analysis.** Image pre-processing were performed by using AFNI toolbox (<http://afni.nimh.nih.gov>)(6). For rs-fMRI data, images were aligned to the space of one specific WT mouse for further group analysis. The first 10 TRs were discarded for having the signals in steady state. Analysis procedures including de-spike, slice timing, head motion correction, linear trend removal, censoring (motion and outlier), and in-plane spatial smooth (0.5 mm gaussian kernel) were applied. Bandpass filter (0.01 Hz – 0.1 Hz) was employed to extract the low frequency fluctuations. For DTI data, DTI indices including mean diffusivity, radial diffusivity (RD), axial diffusivity (AD) and fractional anisotropy (FA) were analyzed based on DTI theory (7) and reconstructed by using in-house C program. Image alignments via AFNI toolbox were performed with the b0 image and registered to the space of the same specific WT mouse, and the transform matrices were then applied onto the MD, RD, AD and FA maps to produce aligned DTI index maps.

Regions of interest (ROI) were manually depicted according to Allen mouse brain atlas (https://mouse.brain-map.org), including medial prefrontal cortex (mPFC), accumbens (ACB), caudate putamen (CPu), cingulate cortex (Cg), insular cortex (Ins), central medial thalamic nucleus (CM), temporal association cortex (TeA), hippocampus (HP), dorsal dentate gyrus (dDG), ventral dentate gyrus (vDG). These specific regions were selected to evaluate the resting-state connection among the targeted regions in this mouse model as well as the connections among common resting-state hubs of mouse brain (8). The DTI indices within these 11 ROIs were also evaluated to assess the microstructural alterations.

Resting-state connectivity among these areas were assessed by calculating the correlation coefficients between each 2 regions, resulting a 11×11 correlation matrix in each rs-fMRI session. Correlation coefficients were averaged through Fisher’s z-transform to generate the averaged correlation matrix in each group, while the difference between the two groups was assessed by two-sample t-test on the z-transformed correlation matrices. The amplitude of low frequency fluctuations (ALFF)(9) were evaluated to characterize the rs-fMRI activity in WT and KO mice. For each mouse, voxel-wise ALFF map was calculated and normalized by whole brain mean. The difference of ALFF between the two groups were evaluated in a voxel-wise manner. All post-processing analysis were performed on Matlab (MathWorks, MA, USA).

**References**

1. Liu WW, Chen SY, Cheng CH, Cheng HJ, Huang PH. BLM-s, a BH3-only protein enriched in postmitotic immature neurons, is transcriptionally upregulated by p53 during DNA damage. *Cell Rep* 2014; **9**:166-179.

2. Hung YF, Chen CY, Shih YC, Liu HY, Huang CM, Hsueh YP. Endosomal TLR3, TLR7, and TLR8 control neuronal morphology through different transcriptional programs. *J Cell Biol*. 2018; **217**:2727-2742.

3. Sheline YI, Mittler BL, Mintun MA. The hippocampus and depression. *Eur Psychiatry* 2002; **17**:300-305.

4. Wray NR, Ripke S, Mattheisen M, Trzaskowski M, Byrne EM, Abdellaoui A, et al. Genome-wide association analyses identify 44 risk variants and refine the genetic architecture of major depression. *Nat Genet* 2018; **50**:668-681.

5. Wei YT, Wu JW, Yeh CW, Shen HC, Wu KP, Vida I, et al. Morpho-physiological properties and connectivity of vasoactive intestinal polypeptide-expressing interneurons in the mouse hippocampal dentate gyrus. *J Comp Neurol* 2021; **529**:2658-2675.

6. Cox RW. AFNI: software for analysis and visualization of functional magnetic resonance neuroimages. *Comput Biomed Res* 1996; **29**:162-173.

7. Basser PJ, Mattiello J, LeBihan D. MR diffusion tensor spectroscopy and imaging. *Biophys J* 1994; **66**:259-267.

8. Liska A, Galbusera A, Schwarz AJ, Gozzi A. Functional connectivity hubs of the mouse brain. *Neuroimage* 2015; **115**:281-291.

9. Zang YF, He Y, Zhu CZ, Cao QJ, Sui MQ, Liang M, et al. Altered baseline brain activity in children with ADHD revealed by resting-state functional MRI. *Brain Dev* 2007; **29**:83-91.

**Supplementary Figure Legends**

**Supplementary Fig. S1. Anatomical and histological analysis of *Blm-s^-/-^* mice**

**A.** *Blm-s^-/-^* (KO) mice are grossly normal and follow Mendelian inheritance pattern. **B** and **C.** The anatomy and histology of KO internal organs are not different from those of *Blm-s^+/+^* (WT) mice. **D** and **E.** The brain/body ratio and brain size at various postnatal (P) day age are similar between WT and KO mice. **F** and **G.** The cortical layer development is normal in the early postnatal KO brain. **H.** Consistent with previous finding (1), embryonic KO cortical neurons are resistant to cell death induced by γ-irradiation.

**Supplementary Fig. S2. Specific expression of AAV-p*Blm-s*-EGFP in neuronal cells and immunoreactivity of BLM isoforms in the adult brain**

**A.** AAV carrying *Blm-s* promoter-driven EGFP is expressed in NG108 neuroblastoma cells, but not in non-neuronal COS-7 cells that have previously been shown to contain non-detectable *Blm-s* transcript (1). **B.** An anti-BLM-l antibody that can detect both BLM-l and BLM-s show specific staining in the hippocampal principal neurons, which include pyramidal cells in CA3 (**1** and **1’**) and CA1, and granule cells in the dentate gyrus (**2** and **2’**).

**Supplementary Fig. S3. Behavioral evaluation of the *Blm-s^-/-^* mice at 3 months of age**

**A**. Experimental timeline and representative swimming path of *Blm-s^-/-^* (KO) and WT mice for 8 consecutive days in the MWM arena. **B**. In rotarod test, the KO mice remained on the wheel as long time as the WT controls at the initial speed and showed similar motor-learning capacity. There are no significant differences between the KO and WT mice for constant speeds and accelerating speeds (4-40 rpm, Two-way repeated measures ANOVA with Holm-Sidak *post hoc* comparison). *n*=13 KO and 12 WT mice. **C** and **D.** Sensation evaluation by cold plate (**C**) and hot plate test (**D**) and compared between KO and WT mice (Two-tailed Student’s *t*-tests). *n*=8 KO and 8 WT mice. All data are shown with errors as mean ± s.e.m..

**Supplementary Fig. S4. Assessment of spatial memory capability of adult *Blm-s^-/-^* mice**

**A**. Graphic illustration of experimental timeline and arena for reversal Morris water maze test. **B-G**. Both *Blm-s^-/-^* (KO) and *Blm-s^+/+^* (WT) revealed progressive shorter time to find the hidden plate along the advancement of training days (**B**) and spent more time in the target quadrant during probe trial (**E**) before platform reversal. The swimming distance and velocity in each quadrant were similar between KO and WT mice (**C** and **D**). After platform reversal, mice of both genotypes quickly learned the environmental subtle change and adjusted their behaviors for new platform localization (**F** and **G**). Errors are shown as mean ± s.d. *n*=4 WT and 7 KO mice. ****P*<0.001, ***P*<0.01, Two-tailed Student’s *t*-test and Two-way repeated measures ANOVA with Holm-Sidak *post hoc* comparison. **H.** Experimental timeline and diagram depicting memory task for object- place recognition (OPR) and novel-object recognition (NOR). There were no differences between KO and WT mice in the total exploration time for both tests and for the discrimination ratio during the retrieval phase of long-term memory test (performed 7-day after encoding). Errors are shown as mean ± s.e.m. *n*=5 WT and 5 KO mice; Two-tailed Student’s *t*-test.

**Supplementary Fig. S5. MRI image analysis of brain regions in adult *Blm-s^-/-^* mice**

**A.** Graphic illustration of brain regions of interest (ROI) used for rsfMRI and DTI image analysis. **B**. Adult *Blm-s^-/-^* (KO) and age-matched WT mice have similar hippocampal volume evaluated by T2-weighted images. *n*=12 KO and 14 WT 3-to-4 months-old mice, Two-tailed Student’s *t*-test.

**Supplementary Fig. S6. Analysis of potential changes of cultured neurons and electron microscopic ultrastructure in the brain of *Blm-s^-/-^* mice**

**A.** Quantification of dendrite spine density of WT and *Blm-s^-/-^* (KO) cultured embryonic cortical neurons. Data were randomly collected from two independent experiments. n, number of examined dendrites. **B.** Representative electron microscopic images from KO and WT mice. **C.** Quantification of PSD morphology of KO and WT mice in dorsal dentate gyrus (dDG) and ventral dentate gyrus (vDG).

**Supplementary Fig. S7. Electrophysiological evaluation of dentate granule cells in *Blm-s^-/-^* mice compared with age-matched wild-type mice**

**A-D**. Summary of the input resistance (R_in_) (**A**), rheobase (**B**), AP threshold (**C**), and AP peak amplitude (**D**) of *Blm-s^-/-^* (KO) (magenta) and WT (green) GCs from dorsal (dDG) and ventral DG (dDG), respectively. WT-dDG, *n*=31; KO-dDG, *n*=31; WT-vDG, *n*=37; KO-vDG, *n*=41. ***P*<0.01, unpaired *t*-test with Welch’s correction.
