# Supplementary figures and images for: Involvement of a BH3-only apoptosis sensitizer gene Blm-s in hippocampus-mediated mood control
Source: Transl Psychiatry. 2022 Sep 26;12:411. doi: 10.1038/s41398-022-02184-6 (PMC9512807; doi:10.1038/s41398-022-02184-6)

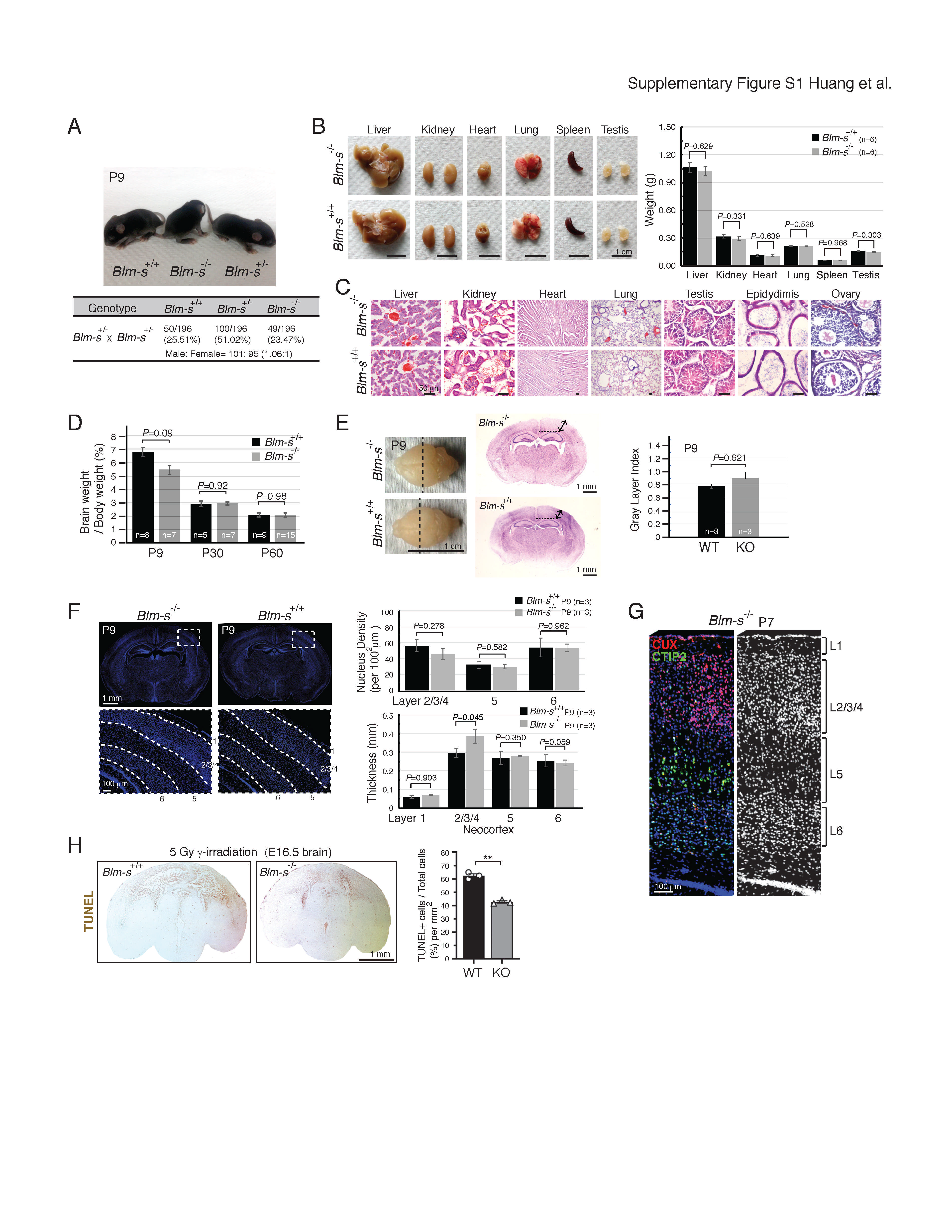

Supplement: Supplementary file 2 — Supplementary Figure S1 [file 41398_2022_2184_MOESM2_ESM.tif]

Supplementary Figure S2 Huang et al.

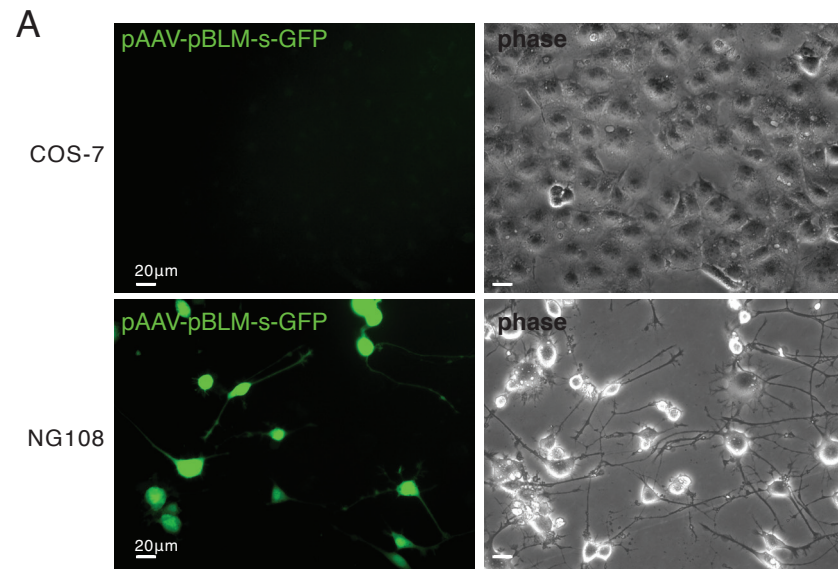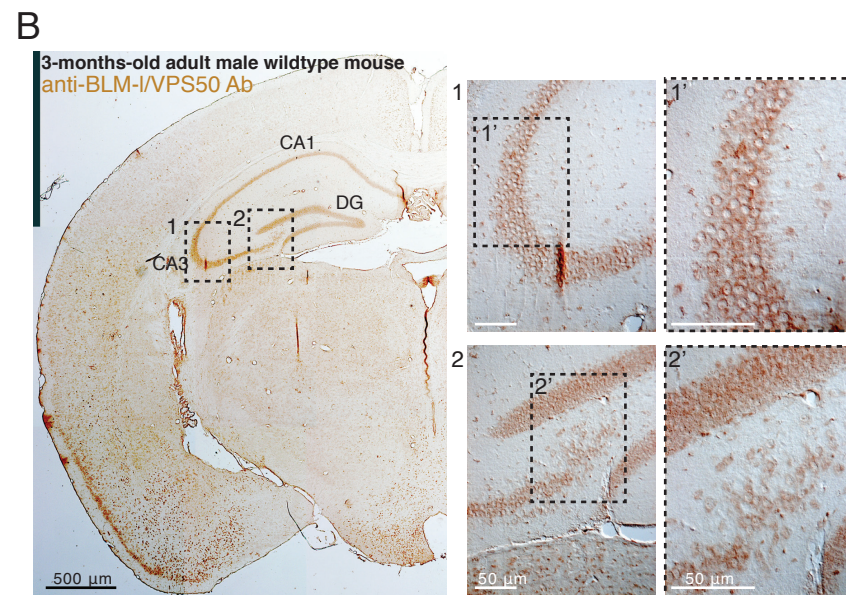

Supplement: Supplementary file 3 — Supplementary Figure S2 [file 41398_2022_2184_MOESM3_ESM.pdf]

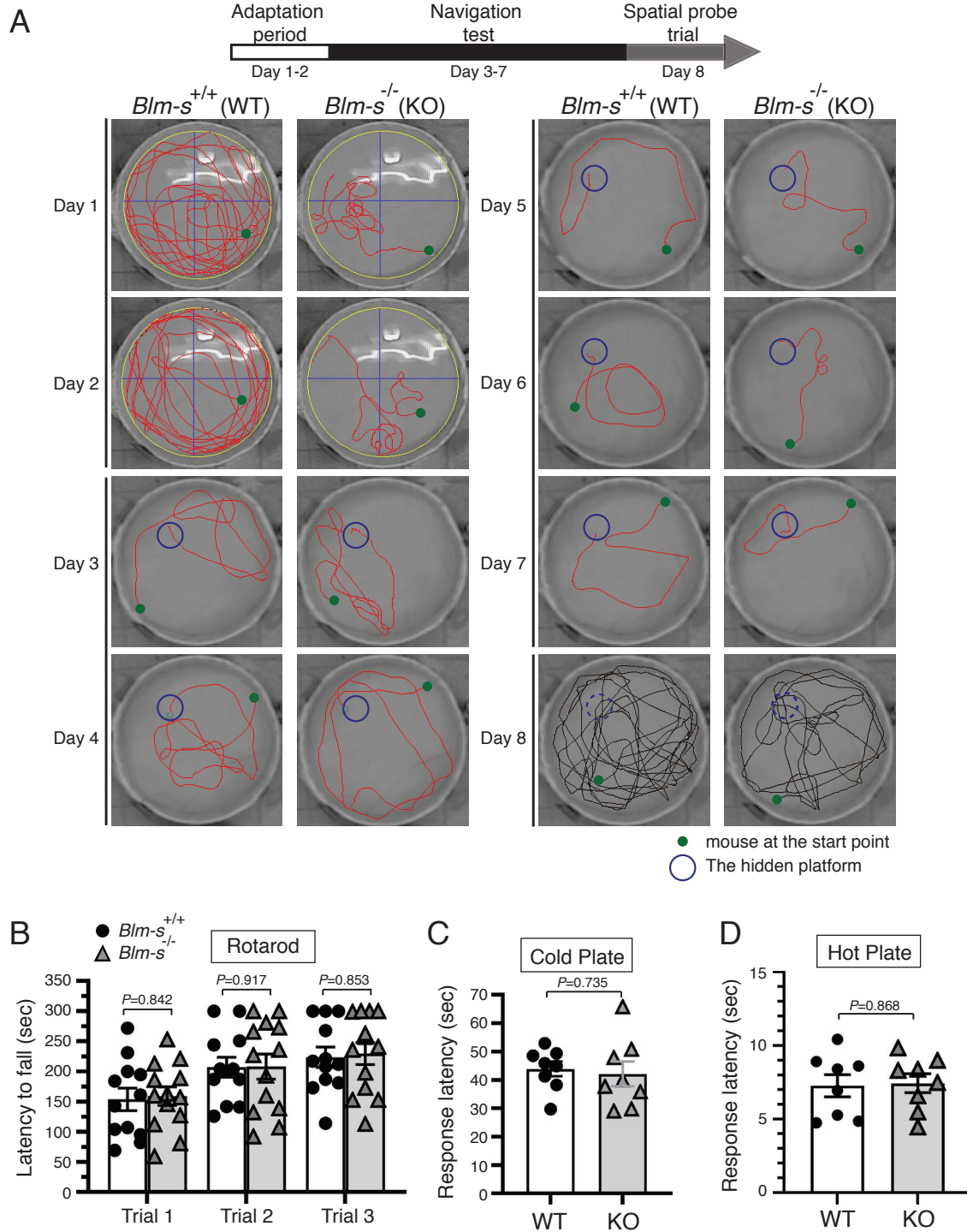

Supplement: Supplementary file 4 — Supplementary Figure S3 [file 41398_2022_2184_MOESM4_ESM.pdf]

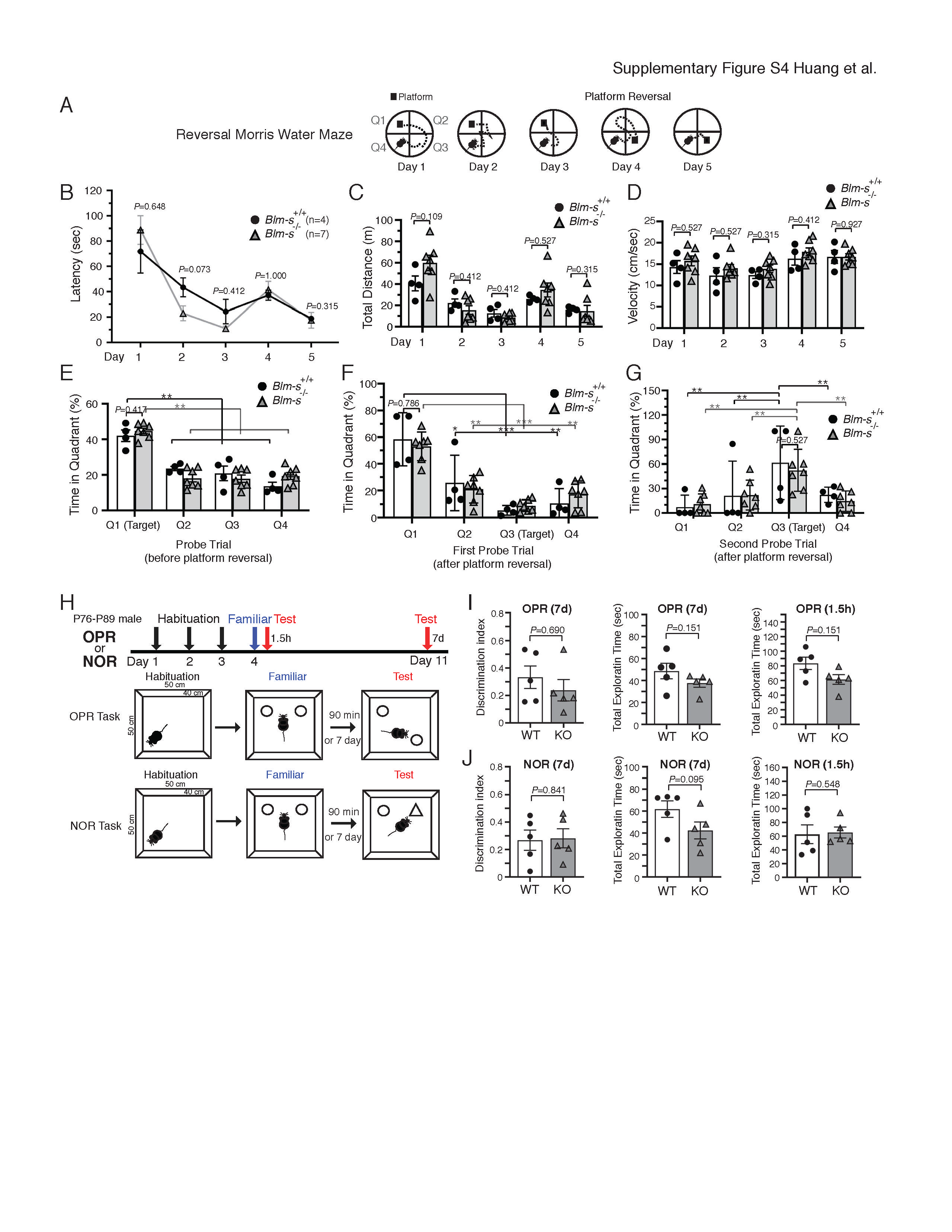

Supplement: Supplementary file 5 — Supplementary Figure S4 [file 41398_2022_2184_MOESM5_ESM.tif]

A

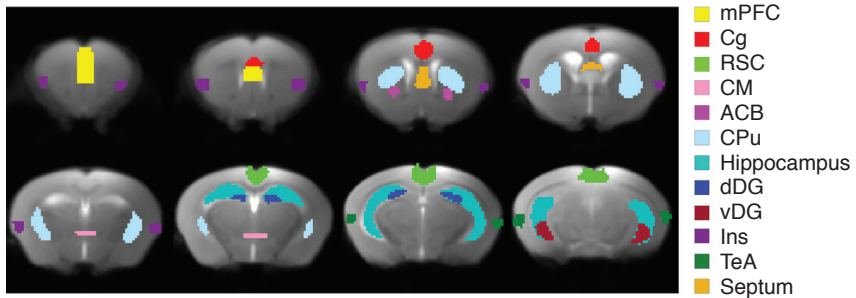

B

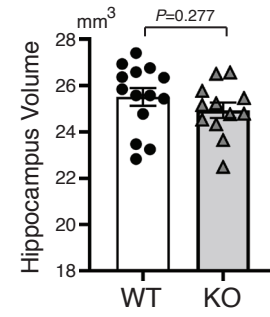

Supplement: Supplementary file 6 — Supplementary Figure S5 [file 41398_2022_2184_MOESM6_ESM.pdf]

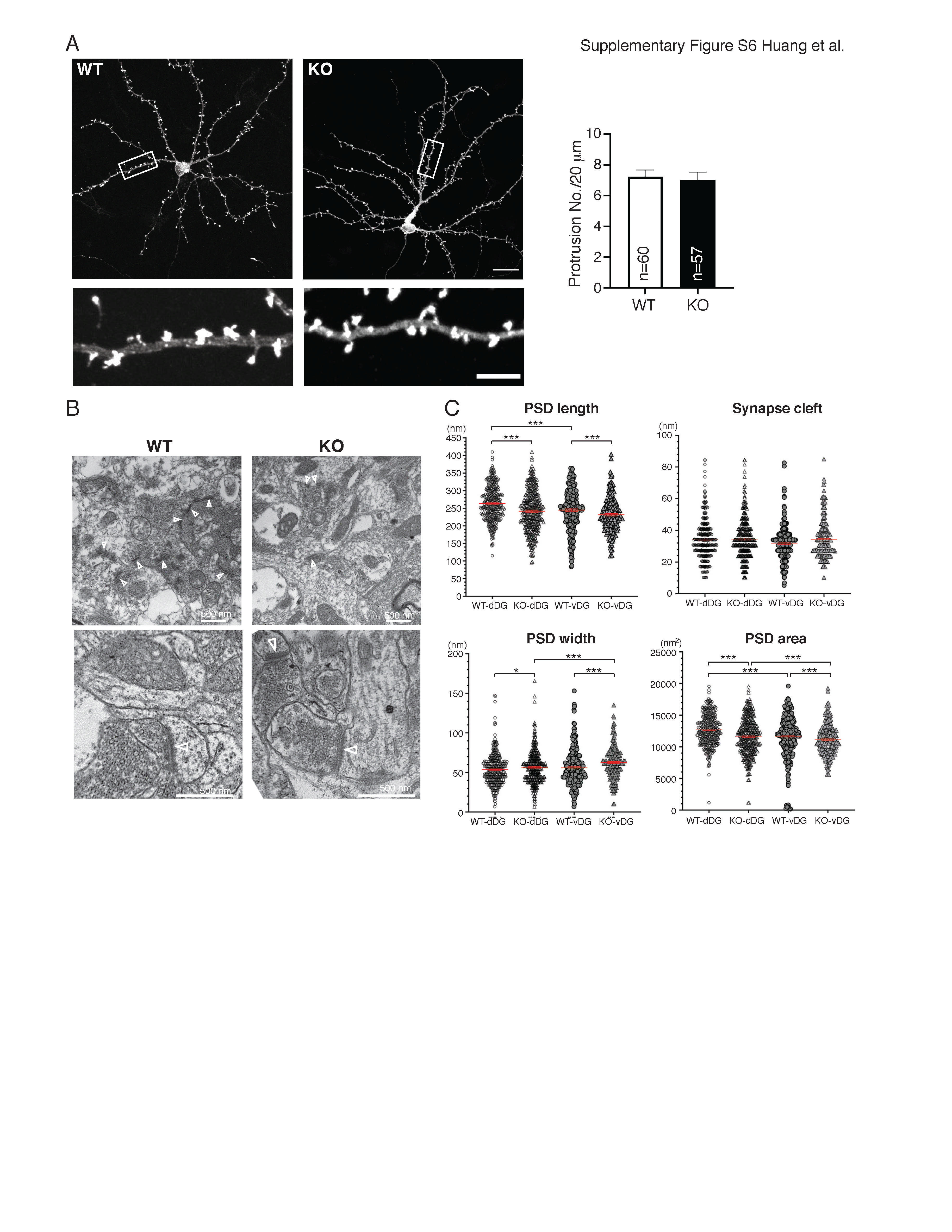

Supplement: Supplementary file 7 — Supplementary Figure S6 [file 41398_2022_2184_MOESM7_ESM.tif]

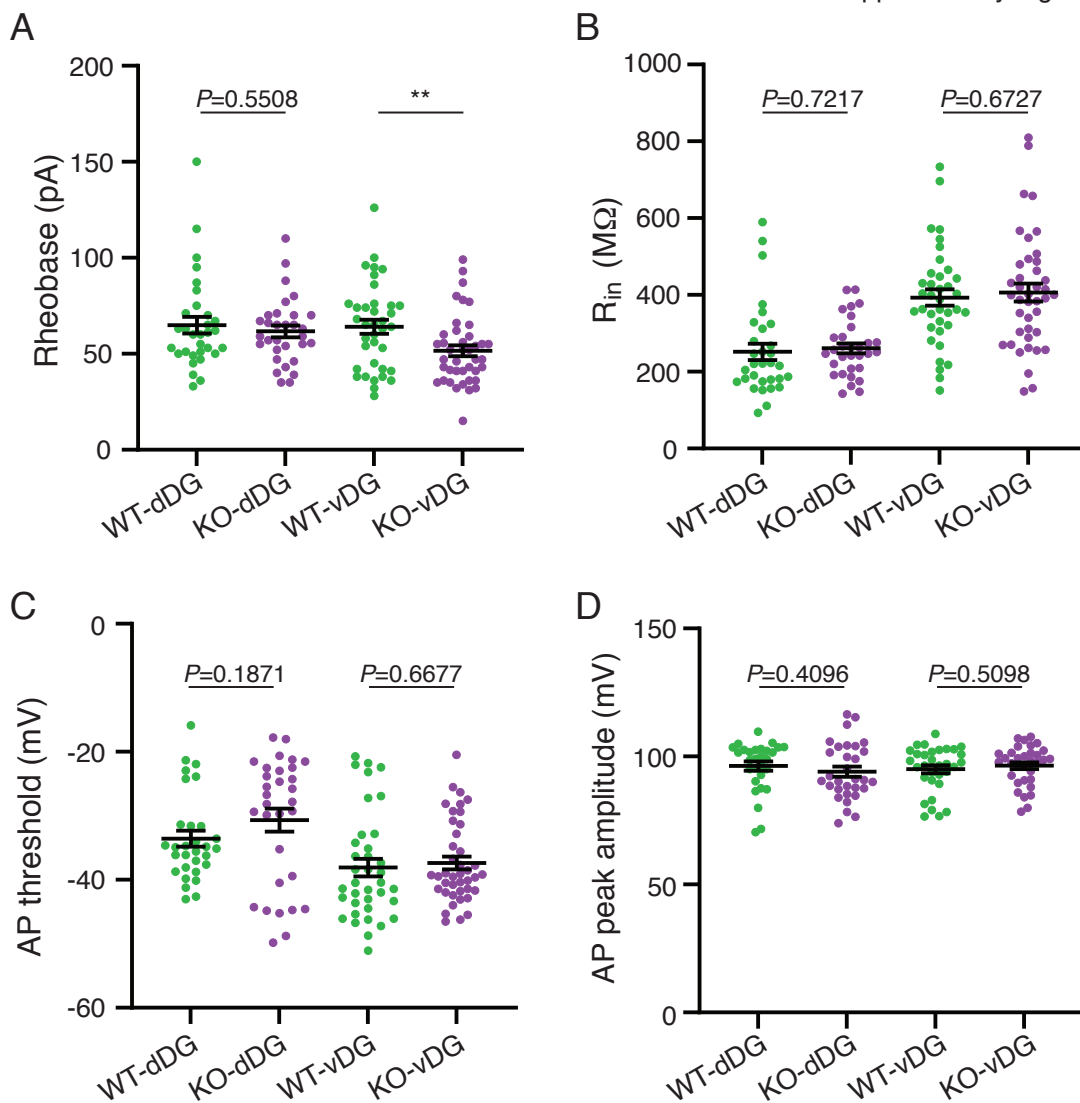

Supplement: Supplementary file 8 — Supplementary Figure S7 [file 41398_2022_2184_MOESM8_ESM.pdf]
